# Ischaemic Preconditioning Suppresses Necrosis of Adipocutaneous Flaps in a Diabetic Rat Model Regardless of the Manner of Preischaemia Induction

**DOI:** 10.1155/2017/4137597

**Published:** 2017-10-18

**Authors:** Christian Ottomann, Markus Küntscher, Bernd Hartmann, Vlado Antonic

**Affiliations:** ^1^Unfallkrankenhaus Berlin, Center for Severe Burn Injuries with Plastic Surgery, Berlin, Germany; ^2^Evangelical Elisabeth Clinic, Berlin, Germany; ^3^Walter Reed Army Institute of Research, Silver Spring, MD, USA

## Abstract

Ischaemic insult in the skin flaps is a major problem in reconstructive surgery particularly in patients with diabetes mellitus. Here, we sought to investigate the effectiveness of ischaemic preconditioning (IP) on diabetic skin flaps in rat animal model. Hundred Wistar rats (90 streptozotocin treated animals and 10 nondiabetic controls) were used. Diabetes mellitus was confirmed by measuring glucose level in blood, HbA1c, and ketonuria. We used blood vessel clamping, hind limb tourniquet, and NO donors (Spermine/NO complex) to induce short-term ischaemia of tissues that will be excised for skin flaps. Animals were followed for 5 days. Flaps were photographed at day 5 and percent of necrosis was determined using planimetry. Significant decrease in percent of necrotic tissue in all groups that received preconditioning was observed. Results show that ischaemic preconditioning suppresses flap necrosis in diabetic rats irrespective of direct or remote tissue IP and irrespective of chemically or physically induced preischaemia. Spermine/NO complex treatment 10 minutes after the flap ischaemia suppressed tissue necrosis. Treatment with NO synthase inhibitor L-NAME reversed effects of IP showing importance of NO for this process. We show that IP is a promising approach for suppression of tissue necrosis in diabetic flaps and potential of NO pathway as therapeutic target in diabetic flaps.

## 1. Introduction

Skin flaps are widely used as a means of definite wound closure, particularly in wounds with large areas of tissue loss. Skin flap necrosis is major problem during the postoperative care and can result in delayed healing/nonhealing wounds and need to revisit surgical procedures. Ischaemia and inadequate blood perfusion of the flaps lead to changes in endothelial cells, excessive tissue edema, and apoptosis resulting in irreversible necrosis of flap tissue. In patients with comorbidities, such as diabetes mellitus, these processes are augmented by underlying pathophysiological conditions. Hyperglycaemic conditions trigger series of cellular and molecular changes that result in unresponsive endothelium, changes in protein structure due to improper glycosylation that translates to impaired blood supply in the tissues, excessive infiltration of immune cells, and increased risk for development of infections [1–3]. Ironically, patients with diabetes often require surgery, skin flap, or skin grafting for definite wound closure and/or limb salvation. Diabetes affects approximately 170 million people worldwide, including 20.8 million in the USA [4] and by 2030 these numbers are projected to double [5] posing enormous burden for healthcare providers, patients, and society as a whole. New therapeutic approaches are needed for improving flap survival and suppression of necrosis.

Ischaemic preconditioning (IP) of skin flap describes a phenomenon where tolerance to ischaemia is induced through provision of a short-term, ischaemia-reperfusion cycle before the actual, longer period of ischaemia, as a result of surgical procedure. The effectiveness of the classical form of ischaemic preconditioning, administered by clamping the vascular pedicle of the flap graft, has been described in several studies [6–8]. Later on, ischaemic preconditioning of flap grafts can also be achieved by administering a short-term ischaemia-reperfusion cycle in regions remote from the flap [9, 10] and other organs: kidney [11], gut [12], liver [13], and heart [14]. The mechanisms underlying classical and graft-remote ischaemic preconditioning have not yet been fully elucidated. However, some studies suggest that nitric oxide (NO), both released and de novo synthesised, plays an essential role in ischaemic preconditioning in general and skin flaps in particular [15–17]. Endothelial cells have important role in the release and synthesis of NO. Endothelial cells respond to changes in NO by changing blood supply in the tissue, expression of surface proteins for immune cells homing, and expression of NO synthase.

In this study, we aimed to investigate the effects of ischaemic preconditioning on the survival of skin flaps in diabetic rats and to compare several different methods, locations, and the timing of preconditioning/treatment. We evaluated several different methods of preconditioning to include direct skin flap tissue ischaemia/reperfusion and remote hind limb ischaemia as well as therapeutically induced preischaemia using Spermine/NO complex.

## 2. Materials and Methods

### 2.1. Animals

All experiments were carried out on male Wistar rats after the approval from the State Office for Health and Social Affairs, Berlin, Germany. The experiments were carried out at the Animal Experimental Facility at Charité, Universitätsmedizin Berlin, Germany. Surgical procedures were performed using gas inhalation of 5% Isoflurane gas in induction chamber, and thereafter the appropriate level of anaesthesia was maintained using 2-3% Isoflurane via nose cone. The animals were followed for five days after the surgery at which time point they were euthanized. Euthanasia was performed using Isoflurane overdose and thoracotomy with excision of the heart according to the “AVMA Guidelines for the Euthanasia of Animals: 2013 Edition.”

### 2.2. Induction of Diabetes Using Streptozotocin

Diabetes was induced by intravenous administration of streptozotocin (N-[methylnitrosocarbamoyl]-D-glucosamine, STZ) at a dose of 65 mg/kg injected into the tail vein. STZ solution was prepared by dissolving STZ in citrate-phosphate buffer (pH 5.0) immediately before use. A single dose regimen (1 × 65 mg/kg body weight) was sufficient to induce diabetes in STZ treated rats.

### 2.3. Measurement of Blood Glucose and HbA1c Levels

Diabetic metabolic status was confirmed by measuring blood glucose and HbA1c levels. These were measured one week after STZ administration and then at four-week intervals until the end of the experiment. In addition, for further verification of diabetic status, ketonuria was determined in the rat's urine using Rothera and Legal's procedure.

### 2.4. Macroscopic and Histopathological Confirmation of the Induction of Diabetes Mellitus

At time of euthanasia tissue was collected for macroscopic evaluation performed by pathologist blinded for the study groups and treatments for the histological confirmation of diabetes mellitus. Briefly, formalin-fixed, paraffin-embedded samples of liver, kidney, eye, heart, pancreas,* vena cava caudalis* and* cranialis*, and* aorta abdominalis *were sectioned at 5 *μ*m and stained using Hematoxylin and Eosin staining. Stained tissues were examined by the experienced pathologist blinded for the study groups and treatments.

### 2.5. Surgical Procedures

After 12 weeks of diabetes, the operation was then carried out in the form of an adipocutaneous flap graft surgical procedure under anaesthesia. The flap graft size was 4 × 10 cm for all animals. The flap graft was raised in pedicular form at the left caudal superficial epigastric artery and vein. The right caudal superficial epigastric artery and vein and the right and left cranial epigastric arteries and veins were ligated. Ischaemic preconditioning was then carried out for three hours using the different procedures described below. At the end of the procedure, the flap was placed on a silicone film and sutured in place to prevent any neo- and revascularisation from the wound base.

### 2.6. Experimental Groups

Experiments were conducted in two stages. In stage I, we used physical means to induce the presurgical ischaemia. Ten healthy nondiabetic controls and 40 male diabetic rats exhibiting diabetic vascular alterations after 12 weeks of diabetes were divided into five experimental groups. In* Group A* (nondiabetic control group, *n* = 10) adipocutaneous flap grafting was performed with three hours of ischaemia administered by clamping the vascular pedicle (left caudal epigastric vein and artery) using a Yasargil clip set at 65 g of compression in nondiabetic rats. In* Group B* (diabetic control group, *n* = 10), a three-hour period of ischaemia was induced in the flap graft by clamping the vascular pedicle (left caudal superficial epigastric artery and vein) with a Yasargil clip set at 65 g of compression in rats with induced diabetes. In* Group C* (flap-adjacent ischaemic preconditioning, *n* = 10) a short-term clamping of the vascular pedicle (left caudal superficial epigastric artery and vein) was performed using a Yasargil clip set at 65 g of compression for 10 minutes. This was followed by 30 min of reperfusion before the actual three-hour period of ischaemia. This group was used to answer the question whether a flap-adjacent ischaemic preconditioning reduces the percentage area of necrosis within an adipocutaneous flap graft in diabetic rats. In* Group D* (flap-remote ischaemic preconditioning, *n* = 10), the contralateral femoral artery (right femoral artery) was surgically exposed before it was clamped for 10 minutes with a Yasargil clip set at 65 g of compression. Afterwards, the femoral perfusion zone was reperfused for 30 minutes before the three-hour period of ischaemia was administered in the flap. In* Group E* (noninvasive, remote tissue, ischaemic preconditioning, *n* = 10), flap-remote ischaemic preconditioning was induced not by an invasive measure, but rather by a tourniquet (Martin®, Tuttlingen, Germany). A ten-minute application of a tourniquet to the right, contralateral hind leg was carried out which was followed by 30 minutes of reperfusion before the three-hour period of flap ischaemia was instigated. The ischaemia of the hind legs of the animals was monitored by pulse oximetry and Doppler sonography. This group served to answer the question whether ischaemic preconditioning can also be instigated noninvasively in type 2 diabetes mellitus.

In stage II of the protocol, we used another 50 diabetic animals and divided them into 5 groups. The animals of* Group F* (pharmacologically induced IP using the nitric oxide donor Spermine, *n* = 10) received 500 nmol/kg BW of the nitric oxide donor Spermine/nitric oxide complex (Sper/NO, Sigma-Aldrich®, Deisenhofen, Germany, chemical name: N-[4-[1-(3-aminopropyl)-2-hydroxy-2 nitrosohydrazino]butyl]-1,3-propandiamine, molecular formula C_10_H_26_N_6_O_2_) intravenously ten minutes prior to clamping the vascular pedicle (left caudal superficial epigastric artery and vein) of the adipocutaneous flap graft using a Yasargil clip set at 65 g of compression. The animals in* Group G* (nitric oxide synthase blocker L-NAME, *n* = 10) received 10 mg/kg of the nonspecific blocker of nitric oxide synthases NO-nitro-L-arginine methyl ester (L-NAME, Sigma-Aldrich, Deisenhofen, Germany) intravenously ten minutes prior to a short-term ten-minute clamping of the vascular pedicle (left caudal superficial epigastric artery and vein) of the adipocutaneous flap graft. This was then followed by thirty minutes of reperfusion of the graft before a three-hour period of ischaemia. This group served to answer the question whether the effect of invasive ischaemic preconditioning can be eliminated by applying a nitric oxide blocker. In* Group H* (nitric oxide synthases blocker L-NAME + tourniquet, *n* = 10), 10 mg/kg L-NAME was administered intravenously before a ten-minute tourniquet ischaemia was applied to the contralateral right hind leg of the rat. The hind leg of the rat was then reperfused for 30 minutes, followed by a three-hour flap graft ischaemia performed by clamping the vascular pedicle (left caudal superficial epigastric artery and vein) using a Yasargil clip set at 65 g of compression. This group served to answer the question whether the effect of noninvasive IP can be eliminated by nitric oxide synthase blockade.

In* Group I* (30 min preischaemic nitric oxide donor application, *n* = 10), 500 nmol/kg BW of Sper/NO was injected intravenously 30 min before the three-hour flap graft ischaemia. The flap graft ischaemia was administered by clamping the vascular pedicle with a Yasargil clip set at 65 g of compression. This group served to determine how preischaemic application of a nitric oxide donor influences the necrosis rate. In* Group J* (postischaemic nitric oxide donor application, *n* = 10), the same dose of Sper/NO (500 nmol/kg) was injected at the end of the three-hour flap ischaemia 5 min before the reperfusion phase. The flap graft ischaemia was administered by clamping the vessel stem with a Yasargil clip set at 65 g of compression.

### 2.7. Evaluation of the Area of Necrosis

The percentage necrosis rate in the adipocutaneous flap graft was assessed by digital photography. Images were evaluated using planimetry software Histo Version 3.0 (Analysis of Histological Images®) of the University of Heidelberg. The absolute and relative areas of necrosis were measured and calculated using pixel numbers.

### 2.8. Statistical Evaluation

The percentage area of necrosis of the adipocutaneous flap grafts is given as the mean ± standard deviation. Statistical evaluation was carried out using analysis of variance (ANOVA) with Dunnet post hoc analysis for comparisons to control group. A *p* value of less than 0.05 was considered to represent statistical significance. The analyses were carried out using SPSS software (version 8.0, MSU, Ann Arbor, MI, USA).

## 3. Results

### 3.1. HbA1c and Blood Glucose Values

For healthy rats the HbA1c value ranges between 4% and 6% and stayed constant through entire length of the study Group A (animals that were not exposed to STZ). Animals exposed to STZ, Groups B to E, combined showed physiological values of HbA1c at week 1 (4.9%) and pathophysiological values thereafter 4 weeks (7.6%), 8 weeks (9.15), and 12 weeks (10.4%.)

**Figure 1:**
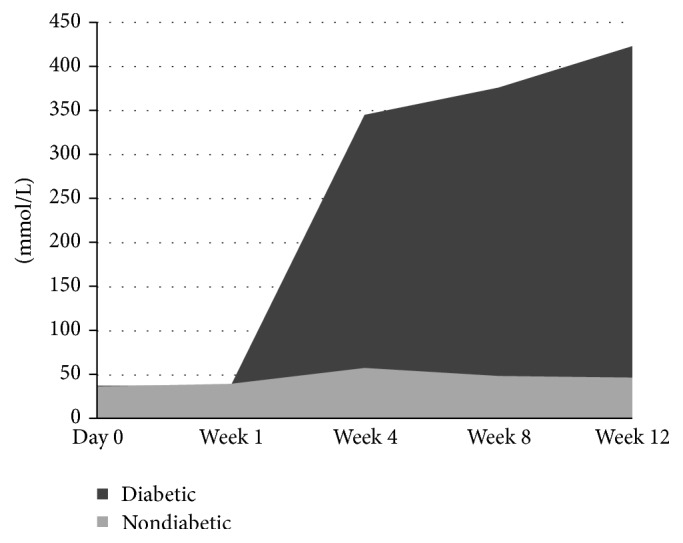
Glucose levels (mmol/L) in STZ treated and nontreated controls. There is a significant increase in the glucose levels in diabetic animals when compared to healthy controls.

Blood glucose level in STZ treated animals steadily increased from week 1 through week 8. Both STZ treated and nontreated controls had starting levels of glucose at 38 mmol/l and 39 mmol/l. Healthy controls at weeks 1, 4, 8, and 12 had blood glucose levels of 40 mmol/l, 58 mmol/l, 49 mmol/l, and 47 mmol/l, respectively. In STZ treated animals levels increased from 38 mmol/l on week 1, to 345 mmol/l on week 4, 376 mmol/l on week 8, and 423 mmol/l on week 12 (Figure 1).

### 3.2. Histological Findings

Histological evaluation performed by the blinded pathologists confirmed the presence of the diabetes mellitus and histopathological changes in the examined organs (data not presented). The changes in the pancreatic islets were mild indicating at least partial functional tissue. The changes in the kidney were severe and show chronic inflammatory as well as degenerative changes. The basement membrane changes are compatible with a severe diabetes mellitus. The retina shows degenerative changes which is compatible with a diabetic retinopathy. Epidermal atrophy was noted with a reduced* stratum spinosum* layer. There was high evidence of morphological changes (atherosclerosis) within the arteria and vena* epigastrica*. In summary these changes were compatible with morphologic endpoints of a type 2 diabetes mellitus.

### 3.3. Ischaemic Preconditioning Reduces Skin Flap Necrosis Irrespective of the Type of Ischaemic Preconditioning

**Figure 2:**
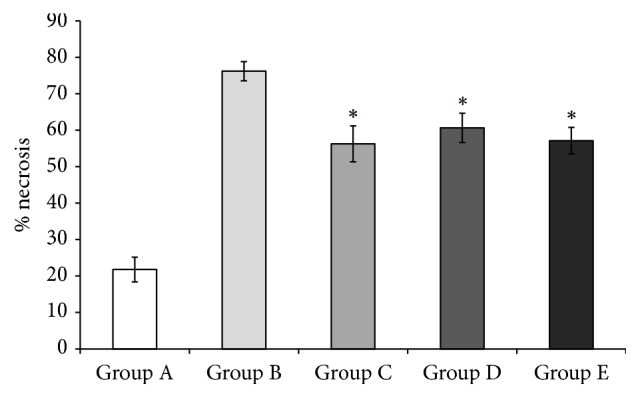
The mean necrosis rate at day 5 after the surgical procedures. There was significant increase in the tissue necrosis in all STZ treated animals when compared to healthy controls (Group A, 21.7 ± 3.4%). Group B: STZ induced diabetic controls (76.2 ± 2.6%); ischaemic preconditioning suppressed necrosis in the diabetic animals and is decreased significantly to Group C: flap-adjacent ischaemic preconditioning (56.3 ± 4.9%); Group D: flap-remote ischaemic preconditioning (60.6 ± 4.0%); Group E: noninvasive, remote tissue, ischaemic preconditioning tourniquet (57.1 ± 3.66%). Results are presented as mean ± SEM. ^*∗*^*p* < 0.05 was considered statistically significant.

Diabetes mellitus significantly contributes to necrosis of ischaemic skin flaps as illustrated by significantly higher percent of flap necrosis when comparing healthy animals (Group A) to all other STZ treated animals after three-hour ischaemia of the adipocutaneous flap grafts (Figure 2). Nondiabetic, control Group A showed an average necrosis rate of 21.7 ± 3.4%. The STZ induced diabetic, control Group B showed an average necrosis rate of 76.2 ± 2.6%. The necrosis rate of the adipocutaneous flap graft in the diabetic animals was significantly reduced by the flap-adjacent ischaemic preconditioning (Group C). In Group C, an average area of necrosis of 56.3 ± 4.9% was comparable with Group B. In Group D, ischaemic preconditioning was induced at the site remote to the skin flap and showed significant protective effect measured by reduced area of necrosis (60.6 ± 4.0%) when compared to diabetic control Group B. In Group E, ischaemic preconditioning using tourniquets necrosis area of 57.1 ± 3.66% was significantly reduced in comparison to the diabetic animals with no ischaemic preconditioned. Ischaemic preconditioning, irrespective of the way of the induction of the short, transient preconditioning, suppressed skin flap necrosis.

**Figure 3:**
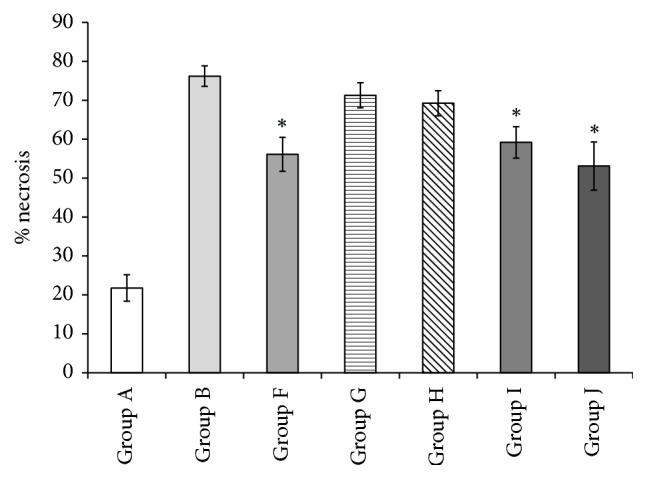
Noninvasive IP suppressed tissue necrosis in diabetic skin flaps. Group A: healthy controls (21.7 ± 3.4%); Group B: STZ induced diabetic controls (76.2 ± 2.6%); Group F: pharmacologically induced IP using the nitric oxide donor Spermine (56.1 ± 4.3%); Group G: nitric oxide synthase blocker L-NAME (71.3 ± 3.2%); Group H: nitric oxide synthases blocker L-NAME + tourniquet (69.25 ± 3.2%); Group I: 30 min preischaemic nitric oxide donor (59.2 ± 4.1%); Group J: postischaemic nitric oxide donor (53.1 ± 6.2%); results are presented as mean ± SEM. ^*∗*^*p* < 0.05 was considered statistically significant.

Isolated single application of the nitric oxide donor Group F resulted in a statistically significantly reduced area of necrosis in the adipocutaneous flap graft when compared to diabetic controls Group B (56.1 ± 4.3% versus 76.2 ± 2.6%, resp.) (Figure 3). Group G served to answer the question whether the reduced necrosis gained by invasive ischaemic preconditioning could be abolished by applying a nitric oxide blocker. The mean area of necrosis in Group G was not statistically different compared to diabetic controls (71.3 ± 3.2% versus 76.2 ± 2.6%, resp., *p* > 0.25) indicating that ischaemic preconditioning is at least in part driven by the production of NO. Ischaemic preconditioning with tourniquet applied on the hind limb of the rats in Group H, which was shown to successfully suppress necrosis (results in Group E), was reversed by application of L-NAME, inhibitor of NO synthase. The average necrosis rate in Group H was not statistically different from the diabetic controls Group B (69.25 ± 3.2% versus 76.2 ± 2.6%, *p* > 0.10, resp.).

Finally, we wanted to evaluate if even earlier application of NO donor (Spermine/nitric oxide complex) has more beneficial effects and if the complex could be applied as treatment after the surgical intervention. The diabetic rats treated with the nitric oxide donor Spermine 30 minutes before the three-hour flap graft ischaemia (Group I) showed a statistically significant reduction in mean necrosis of 59.2 ± 4.1% versus 76.2 ± 2.6% seen in the diabetic control group (Group B). Importantly, the Spermine/nitric oxide complex was successful in reducing necrosis in diabetic mice when applied 10 minutes after the ischaemia (Group J). Group J had a statistically significant lower percent of necrotic tissue (53.1 ± 6.2%) when compared to Group B (76.2 ± 2.6%), *p* < 0.05.

## 4. Discussion

In the present study, we have shown that ischaemic preconditioning is a successful pretreatment for suppression of necrosis in the skin flaps of diabetic rats. This suppression was irrespective of the preischaemia location: direct flap preconditioning or remote hind limb preconditioning. We then tested if NO donors could be used as treatment and applied the Spermine/NO complex as pretreatment and treatment and showed that suppression of necrosis is independent of the timing of application. These effects were reversed by application of NOS inhibitor confirming that IP exerts its beneficial effects at least partially through NO pathway.

Ischaemic preconditioning is the induction of ischaemia tolerance by preadministering a defined period of ischaemia in order to reduce any later occurring ischaemic reperfusion injury within a tissue. We evaluated several different methods of preconditioning to include direct skin flap tissue IP and remote hind limb IP in diabetic rats. Existing studies regarding the effects of IP on skin flaps showed that in normal physiological conditions this approach has therapeutic potential [18–21]. These studies are conducted in healthy rats, in the absence of decreased responsiveness of the endothelium. To the best of our knowledge, studies on the effects of IP on adipocutaneous flaps, especially within the context of comorbidities such as diabetes, have not been published until now. Limited literature exists about the effects of diabetes mellitus on success of IP on other organs. Galagudza et al. concluded in their study of myocardium of diabetic rats that the effects of IP are attenuated in diabetic rats [22]. It should nevertheless be said that the comparability of the data is limited since the animals had only been manifesting the diabetes for a period of six weeks [22]. Wang et al. on the other hand showed that diabetes mellitus blocks the effects of ischaemic preconditioning on rat heart [13]. Their group was able to demonstrate that chronic hyperglycaemia reversed the cardioprotective effect of IP after myocardial infarction. The cause for this was assumed to be damage to the endothelium. Our results support the findings that IP suppresses tissue necrosis in skin flaps, in general, and for the first time, in diabetic rats in particular. We demonstrated that even remote IP significantly protects tissue (Group E 57.1% versus Group B 76.2%) which can potentially have significant impact on patient preparations for surgery.

In the skin flaps/grafts, after the blood supply is completely interrupted, complex cellular and intracellular reaction cascades are triggered [23, 24]. As a result of the ischaemia there is a disruption of electrolyte equilibrium, which culminates in metabolic acidosis, cellular reconstruction, and ultimately apoptosis resulting in irreversible tissue destruction [25, 26]. From the teleological point of view, the tissue outcome depends on the successful and harmless reperfusion of the tissue, in which endothelial cells and NO pathway have key regulatory role. Numerous studies have demonstrated tissue edema [27, 28], depending on the NO release and its de novo synthesis [29]. In the prolonged hyperglycaemic conditions, the oversaturation with sugars induces disbalance in the endothelial functions. Typically, endothelium-mediated vasodilatation is attenuated, which otherwise would represent an important adaptation mechanism to reperfusion [30, 31]. There are a number of experimental findings that have demonstrated a link between insulin resistance and endothelial dysfunction. Steinberg et al. demonstrated that the vasodilatory effect of the muscarinic receptor agonist methacholine is reversed in diabetic animals, while endothelium independent vasodilation induced by sodium nitroprusside remains unchanged [32]. Montagnani et al. were able to demonstrate that nitric oxide-dependent endothelial vasodilatation is disrupted in the insulin-resistant animal [33, 34]. Therefore, we conducted series of experiments to determine if different pretreatment regimens with NO donor (30-minute preconditioning with Spermine/NO complex) and treatment (Spermine/NO applied 10 minutes after the surgery) could decrease necrosis in diabetic flaps. Indeed, disbalance in NO release/synthesis was responsible for increase in tissue necrosis seen in diabetic animals (Group B). This process was significantly suppressed after the application of NO donors, either as a pretreatment or as treatment. Importantly, when the animals were exposed to NOS inhibitor, protective effects of both remote preconditioning and treatment with Spermine/NO complex were diminished. Expression of endothelial NOS has a positive feedback loop, leading to overall increase in concentration of NO in the tissue [15]. Our group previously demonstrated this in flaps [16] and muscle [17]. Indeed, when NOS inhibitor was applied, there was significant suppression of protective effects of IP.

Limitations of this study were that we present limited mechanistic data for the support of IP in diabetic animals. Nevertheless, the results clearly show the protective effects of IP. We are currently conducting studies that will address mechanistic questions and with this study present proof-of-concept results.

## 5. Conclusions

In summary, the present study shows that ischaemic preconditioning is protective in skin flap in type 2 diabetes mellitus rats. Both invasive flap-adjacent IP at the graft pedicular vessel as well as with flap-remote IP of the contralateral femoral artery significantly reduced area of necrosis compared to diabetic controls. A noninvasive ischaemic preconditioning using NO donors is successful both as treatment and pretreatment. Finally, these processes are dependent on the NO pathway and grant further investigations.
